# Incidence of Alopecia and Hair Loss Among Female Active Component Service Members, 2010–2022

**Published:** 2024-09-20

**Authors:** Alexis A. McQuistan, Thomas Wilkerson, Sithembile L. Mabila

**Affiliations:** 1 Epidemiology and Analysis Section, Armed Forces Health Surveillance Division, Defense Health Agency, Silver Spring, MD

## 1 BACKGROUND

Alopecia, or hair loss, can have several different underlying causes and is typically classified as a scarring or non-scarring condition.^1^ In non-scarring alopecia, there is potential for hair regrowth, while in scarring alopecia hair follicles are irreversibly destroyed, leading to permanent hair loss.^2,3^ Cicatricial alopecia, or scarring hair loss, is the result of permanent damage to hair follicles. Androgenic alopecia is hair loss caused by the effects of androgens on hair follicles, and is the most common cause of non-scarring hair loss among both men and women. Alopecia areata is non-scarring hair loss caused by an autoimmune disorder, with a lifetime prevalence ranging from 1.7% to 2.1% based on the Rochester Epidemiology project, using data from 1990 to 2009.^4^ Other non-scarring hair loss can be caused by disruptions to the hair cycle, as in the case of telogen effluvium, in which a physiological or emotional stressor causes sudden hair loss, as well as anagen effluvium, in which impairment of mitotic or metabolic activity of the hair follicle leads to abrupt hair loss.

Hair loss is also caused by repeated hair follicle damage by external causes. Traction alopecia is hair loss caused by repeated pulling of the hair, often caused by taut or tight hairstyles.^5^ Traction alopecia can be non-scarring or scarring, depending on the extent of hair follicle damage caused by hair styling. Female active component service members (ACSMs) are at risk for hair loss due to traction alopecia, as military grooming standards often required hairstyles such as tight ponytails, buns, or braids over a prolonged period of time.^6^ The U.S. military’s recent efforts to change grooming standards have been driven by the desire to recognize diversity in the military and address grooming-related health concerns.^7^

Estimates of traction alopecia among service members have been difficult, as there are no specific diagnosis codes for traction alopecia in the International Classification of Diseases, 9th and 10th revisions (ICD-9/ICD-10). A prior study on the prevalence of alopecia among female ACSMs between 2010 and 2019 reported that 2.7% of female ACSMs had traction alopecia diagnoses.^8^ This estimate may not be accurate, as the authors used the ICD-9 code 704.01 (alopecia areata) and ICD-10 code Q84.0 (congenital alopecia),^9^ which are not fully representative of traction alopecia cases in administrative health records.

Despite the difficulty in conducting large studies on traction alopecia among service women, efforts should be made to quantify hair loss issues faced by female service members. The National Defense Authorization Act (NDAA) draft for fiscal year 2024 that passed the House Armed Services Committee includes an amendment to determine the cost of expanding treatments covered by TRICARE for traction alopecia, citing the disproportionate impact on Black female service members’ likelihood to develop hair loss and traction alopecia due to adherence to strict standards of military dress.^10^ The NDAA amendment called for the number of service women with traction alopecia to be determined for cost estimates. Although this cannot be directly measured with administrative health records, a broader look at alopecia and hair loss among female ACSMs can be made. This study aimed to determine the incidence of hair loss among active component service women, describe the types of hair loss, and summarize potential racial and ethnic disparities of hair loss among service women over the last 12 years.

## 2 METHODS

The study population included female ACSMs in service in the U.S. Army, Navy, Air Force, and Marine Corps between January 1, 2010 and December 31, 2022. Data from the Defense Medical Surveillance System (DMSS), maintained by the Armed Forces Health Surveillance Division (AFHSD), were used to obtain demographics and medical encounter data for the study population. Inpatient and outpatient medical encounters at military hospitals and clinics as well as civilian facilities offering private sector care were included. Race and ethnicity were self-reported by service members.

ICD-9/ICD-10 diagnostic codes (ICD-9: 704.0*; ICD-10: L63*, L64*, L65*, L66*) were used to define cases of alopecia. The current study modeled alopecia case definition parameters based on a retrospective case series, in which investigators verified by chart review a high probability of alopecia areata for patients with at least 1 ICD-10 code (L63*).^11^ An incident case of alopecia was defined as 1 medical encounter, either inpatient or outpatient, with a qualifying ICD-9/ICD-10 code in any diagnostic position. The first case-defining encounter was used as the incident date. A case could be counted once per lifetime. Incident cases from 2016 to 2022 were categorized into 4 different categories based on the qualifying ICD-10 code from the incident encounter. Incidence rates for alopecia areata, androgenic alopecia, other non-scarring hair loss, and cicatricial alopecia from 2016 to 2022 were calculated. Incidence rate calculations by alopecia category were limited to ICD-10-coded encounters because ICD-9 coding does not include these categories.

Person-time was calculated for each service member from January 1, 2010 through December 31, 2022. Service members whose case-defining incident encounter in DMSS preceded the start of the surveillance period were excluded. Incidence rates were calculated as incident alopecia diagnoses per 100,000 person-years (p-yrs).

## 3 RESULTS

Between 2010 and 2022, a total of 21,329 active component U.S. service women were diagnosed with some type of alopecia (**Table**). Among ICD-9-coded encounters, 79% of incident encounters were coded with unspecified alopecia (704.00) (data not shown). Twelve percent of ICD-9-coded cases were diagnosed with alopecia areata (704.01). Among ICD-10-coded encounters between 2016 and 2022, 80% of incident encounters were coded with other non-scarring hair loss (L65*) (**Table**). Fourteen percent of ICD-10-coded cases were diagnosed with alopecia areata (L63*). Among ICD-10-coded cases, non-Hispanic Black ACSMs accounted for the largest number of cases, with highest rates of alopecia areata (n=724; 38%), scarring alopecia (n=265; 59%), and the highest rate of other non-scarring hair loss (**Table**). Hospitalizations were included in this analysis, but only 24 cases had an incident encounter in an inpatient setting, and alopecia was not the primary diagnoses for any of the inpatient encounters (data not shown).

The overall incidence rate of alopecia was 804.4 per 100,000 p-yrs (**Table**). Non-Hispanic Black and Hispanic female ACSMs had the highest incidence rates among all races and ethnicities, at 1,138.7 per 100,000 p-yrs and 1,013.6 per 100,000 p-yrs, respectively (**Table**). Non-Hispanic Black female ACSMs were more than twice as likely to be diagnosed with alopecia compared to non-Hispanic White female ACSMs.

Between 2016 and 2022, non-scarring hair loss had the highest rate (734.2 per 100,000 p-yrs) compared to the other 3 categories of alopecia and was a likely driver of the overall rates from 2010 to 2022. Non-Hispanic Black female ACSMs were more than 3 times as likely to be diagnosed with alopecia areata and more than 5 times as likely to be diagnosed with cicatricial alopecia (**Table**). Incidence rates of alopecia increased with age (**Table**), overall as well as by type. Among other demographic categories, women in the 40-44-year age group, in Army service, as well as senior enlisted and health care occupation categories had the highest rates (**Table**).

The incidence rate for alopecia more than doubled from 2010 (564.3 per 100,000 p-yrs) to 2022 (1,228 per 100,000 p-yrs) (**Figure**). Rates for alopecia steadily increased between 2010 (564.3 per 100,000 p-yrs) and 2020 (841.6 per 100,000 p-yrs) (**Figure**) before increasing by 38% between 2020 and 2021 (1,166.5 per 100,000 p-yrs). The sharp increase in rates between 2020 and 2021 was seen within all race and ethnicity groups, although rates for Hispanic women had been increasing since 2019. Non-Hispanic Black women had the highest rates throughout the surveillance period (**Figure**). The incidence rate of alopecia among non-Hispanic Black women increased between 2010 and 2017 (938.6 per 100,000 p-yrs and 1,199.3 p-yrs) before incidence rates began to decline until 2020 (1,078.5 per 100,000 p-yrs).

## 4 DISCUSSION

This study found higher incidence rates of alopecia among non-Hispanic Black and Hispanic female ACSMs, consistent with other studies.^8,12^ Non-Hispanic Black ACSMs represented the largest proportion of cases of alopecia and hair loss overall, and had the highest rates of alopecia areata, scarring alopecia, and other non-scarring hair loss. The frequency of diagnoses for unspecified non-scarring alopecia in female ACSMs may be an indication that cases of traction alopecia are captured with non-specific alopecia codes, but this cannot be confirmed utilizing administrative health records without a validated surveillance case definition.

The hair concerns of female service members have received more attention in recent years. Changes throughout the services have been made, authorizing a wider array of hairstyles and increasing hair bulk limits in an effort to be more inclusive of different hair types.^7^ In 2020, the U.S. Air Force authorized an increase in hair bulk of up to 4 inches, and in 2021 both the Air Force and U.S. Army authorized ponytails and braids.^7^ These changes were brought about by the efforts of service women, such as the Air Force Women’s Initiative Team comprised of volunteer service women,^13^ to implement changes in outdated hair policies. The increased attention to hair health could be driving female ACSMs to seek health care for any hair loss issues and may be the cause for the increase in incident cases seen starting in 2021. Increased awareness in the general public may also continue to drive these trends, driven by high profile celebrities and social media content.^14^

The timing of the observed increase in alopecia incidence in 2021 raises the question if that increase is associated with the COVID-19 pandemic. There is evidence to suggest COVID-19 is associated with telogen effluvium, which can be provoked by stressful events, trauma, illness, and more.^15,16^ There was a 71% increase in this study population of the number of incident cases of telogen effluvium from 2020 to 2021, a difference of 72 cases, and a 41% increase in the other non-scarring alopecia category (L65*) overall (data not shown). Further study is required to determine whether SARS-CoV-2 or stressors during the pandemic were contributing factors to the increase documented.

Evaluating the impact of changes to grooming standards on traction alopecia could not be measured directly in this study, as traction alopecia cannot be identified through ICD-9/ICD-10 codes, a limitation of this study. Additionally, this study did not report the co-occurring or underlying health conditions that may have contributed to non-specific hair loss, such as autoimmune disorders, pregnancy and postpartum hair loss, or thyroid disorders. Further study would be required to better understand cases of unspecified hair loss.

## Figures and Tables

**Table:** Incidence of Alopecia and Hair Loss by Demographic and Military Characteristics, Female Active Component Service Members, 2010–2022

	2010–2022			2016–2022^a^ (n=13,496)							
	All Types, Alopecia or Hair Loss			Alopecia Areata		Androgenic Alopecia		Other Non-Scarring Hair Loss		Cicatrical Alopecia	
	No.	Person-years	Rate^b^	No.	Rate^b^	No.	Rate^b^	No.	Rate^b^	No.	Rate^b^
Total	21,329	2,651,417	804.4	1,906	129.5	338	23.0	10,804	734.2	448	30.4
Race and ethnicity											
White, non-Hispanic	6,283	1,174,195	535.1	392	62.4	122	19.4	3,447	548.8	83	13.2
Black, non-Hispanic	7,497	658,372	1,138.7	724	204.1	84	23.7	3,346	943.2	265	74.7
Hispanic	4,565	450,380	1,013.6	472	168.4	73	26.1	2,500	892.2	58	20.7
Other/unknown^c^	2,984	368,471	809.8	318	152.6	59	28.3	1,511	725.1	42	20.2
Age group, y											
<20	461	207,351	222.3	51	40.9	3	2.4	213	170.8	13	10.4
20–24	4,670	896,814	520.7	447	89.1	54	10.8	2,289	456.4	89	17.7
25–29	5,421	665,129	815.0	490	134.2	77	21.1	2,725	746.4	114	31.2
30–34	4,288	407,627	1,051.9	366	162.7	77	34.2	2,167	963.3	86	38.2
35–39	3,484	262,751	1,326.0	322	218.8	63	42.8	1,917	1,302.6	72	48.9
40–44	1,946	131,636	1,478.3	150	224.5	31	46.4	981	1,468.3	39	58.4
45+	1,059	80,109	1,321.9	80	194.2	33	80.1	512	1,242.7	35	85.0
Service											
Army	8,895	897,458	991.1	808	170.0	153	32.2	4,314	907.7	195	41.0
Navy	4,623	765,192	604.2	471	105.7	71	15.9	2,401	538.7	101	22.7
Air Force	6,634	798,226	831.1	502	113.6	105	23.8	3,480	787.6	117	26.5
Marine Corps	1,177	190,541	617.7	125	115.0	9	8.3	609	560.3	35	32.2
Rank, grade											
Junior Enlisted (E1-E4)	6,668	1,227,772	543.1	639	93.9	75	11.0	3,205	470.8	139	20.4
Senior Enlisted (E5-E9)	10,336	922,132	1,120.9	924	181.3	167	32.8	5,322	1,044.1	239	46.9
Officer (O1-O3, [W1-W3])	2,735	346,320	789.7	241	124.4	52	26.8	1,395	719.8	43	22.2
Senior Officer (O4-O10, [W4-W5])	1,590	155,194	1,024.5	102	117.1	44	50.5	882	1,012.2	27	31.0
Military occupation											
Combat-specific^d^	349	64,018	545.2	37	90.2	6	14.6	195	475.3	4	9.7
Motor transport	544	82,500	659.4	56	118.9	6	12.7	252	535.1	14	29.7
Pilot/air crew	196	39,624	494.6	18	78.2	4	17.4	110	477.9	1	4.3
Repair/engineering	3,192	525,339	607.6	333	111.6	47	15.8	1,615	541.4	71	23.8
Communications/intelligence	8,271	861,248	960.4	741	160.8	119	25.8	4,073	883.9	196	42.5
Health care	4,938	499,009	989.6	377	139.8	99	36.7	2,548	944.5	82	30.4
Other	3,839	579,679	662.3	344	103.8	57	17.2	2,011	606.6	80	24.1

Abbreviations: n, number; No., number; y, years.

^a^2016-2022 date range includes only ICD-10-coded encounters.

^b^Incidence rate per 100,000 person-years.

^c^Includes those of American Indian/Alaska Native, Asian/Pacific Islander, and unknown race or ethnicity.

^d^Infantry/artillery/combat engineering/armor.

**Figure:**
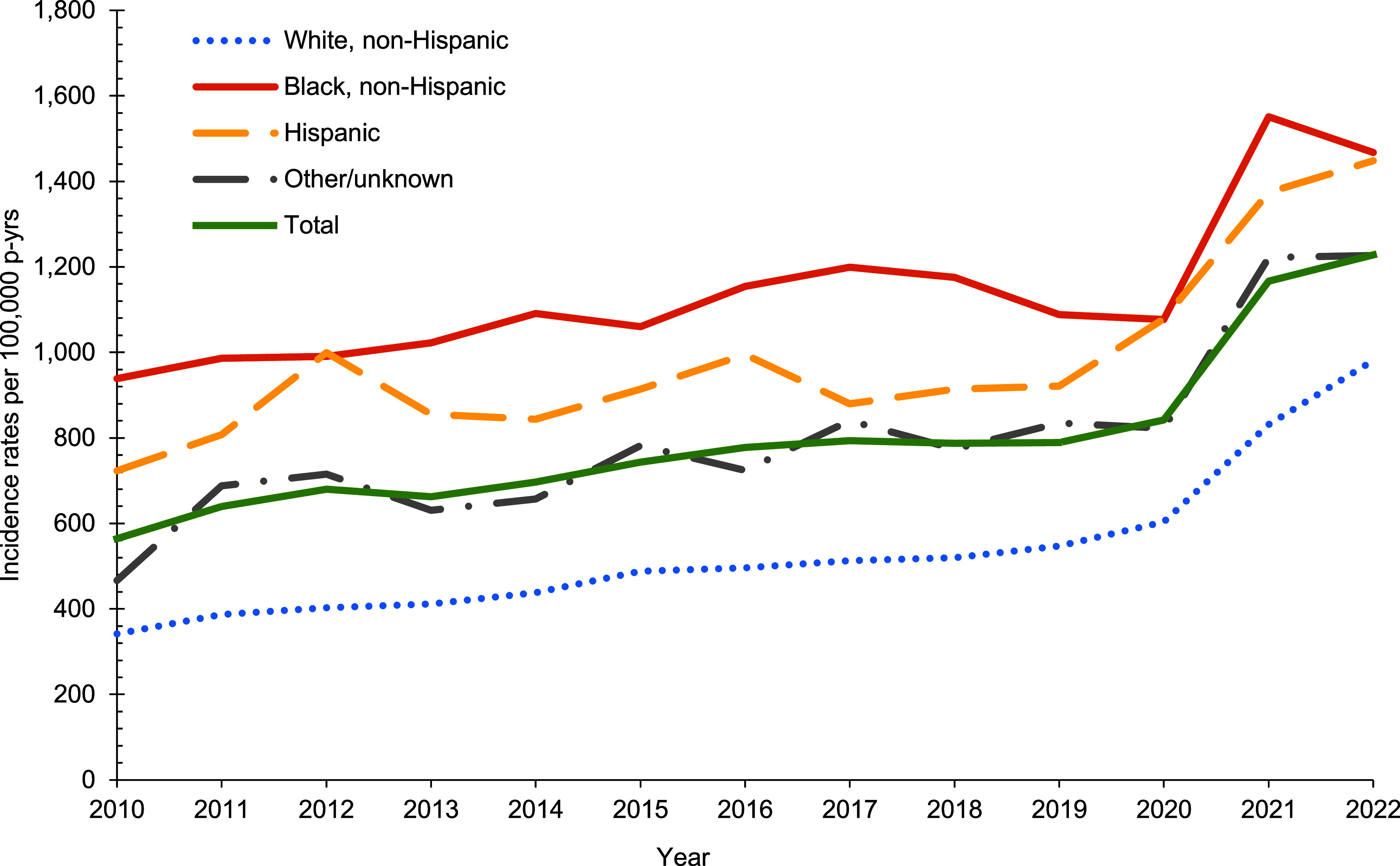
Crude Annual Incidence Rates of Alopecia by Race and Ethnicity, Female Active Component Service Members, 2010–2022

## AUTHORS' AFFILIATION

Epidemiology and Analysis Section, Armed Forces Health Surveillance Division, Defense Health Agency, Silver Spring, MD: Ms. McQuistan, Mr. Wilkerson, Dr. Mabila
